# The accuracy of locating the cricothyroid membrane by palpation – an intergender study

**DOI:** 10.1186/1471-2253-14-108

**Published:** 2014-11-22

**Authors:** Mark Campbell, Hilary Shanahan, Simon Ash, Jonathan Royds, Viera Husarova, Conan McCaul

**Affiliations:** Department of Anesthesia, The Rotunda Hospital, Parnell Square, Dublin 1, Ireland; Department of Anaesthesia, Mater Misericordiae University Hospital, Dublin, Ireland; School of Medicine and Medical Sciences, University College Dublin, Dublin, Ireland

**Keywords:** Airway, Anatomy, Anaesthesia – Emergency, Complications- Airway Obstruction, Complications – Hypoxia, Training

## Abstract

**Background:**

The cricothyroid membrane (CTM) is the recommended site of access to the airway during cricothyrotomy to provide emergency oxygenation. We sought to compare the ability of physicians to correctly identify the CTM in male and female patients.

**Methods:**

In a prospective observational study, anaesthetists were asked to locate the CTM by palpation which was then identified using ultrasound and the distance between the actual and estimated margin of the CTM was measured. Participants assessed the ease of CTM palpation using a visual analog scale. In a second series, the angulation of the posterior junction of the thyroid laminae was measured using ultrasound.

**Results:**

23 anaesthetists and 44 subjects participated. A total of 36 assessments were carried out in each gender. Incorrect identification of the CTM was more common in females (29/36 vs. 11/36, P < 0.001) and the distance from the CTM in the vertical plane was greater (11.0 [6.5–20.0] vs. 0.0 [0.0–10.0] mm, P < 0.001). In females distance from the CTM correlated positively with neck circumference (P = 0.005) and BMI (P = 0.00005) and negatively with subject height (P = 0.01). Posterior thyroid cartilage angulation was greater in females (118.6 ± 9.4° vs. 95.9 ± 12.9°, P = 0.02) and was lower in patients with correctly identified CTMs (100.0 ± 14.9° vs. 115.6 ± 15.9°, P = 0.02). VRS palpation correlated with decreased posterior thyroid cartilage angulation (P = 0.04).

**Conclusions:**

CTM localisation is more difficult in female subjects irrespective of body habitus. It may be prudent to localize this structure by additional means (e.g. ultrasound) in advance of any airway manoeuvres or to modify the cricothyrotomy technique in the event that it is necessary in an emergency.

## Background

Emergency access to the trachea via the cricothyroid membrane (CTM) is a core skill for anaesthetists and constitutes the ultimate rescue manoeuvre to restore oxygenation in failed intubation algorithms. It may also be the primary airway choice in circumstances such as facial trauma [1–4]. Recently published figures from the NAP 4 project in the United Kingdom indicate that this procedure had a failure rate of 64% when performed by anaesthetists [5]. The failure rate was highest for narrow bore cannula (63%) and somewhat lower for wide bore cannula attempts (43%) suggesting that equipment performance may contribute to the outcome. The overall failure rate of 64% is considerably greater than that reported in older published series where the procedure was frequently performed by pre-hospital paramedical personnel [5–8]. The reasons for this discrepancy are unclear. Equipment related issues aside, the success or failure of the procedure is obviously also dependent on the ability of the clinician to correctly identify the CTM in the first instance. Failure to identify this structure may result in serious complications including failure to oxygenate or trauma to adjacent structures in the airway and vasculature [6–10]. The more obvious sources of difficulty in identifying the CTM include obesity or altered neck anatomy, but in many of the patients in whom the procedure failed, risk factors were absent.

A previous study that was conducted by our research group found that misidentification of the cricothyroid membrane is common in female subjects but did not include a direct comparison with males [9]. In the current study which is a follow on from the previously referenced work, we sought to determine if it is more difficult to locate the CTM by palpation in females than males. We used ultrasound to identify the CTM in a group of male and female subjects and compared the ability of Anaesthetists to identify this structure using palpation. On the basis that the female larynx is generally smaller and less prominent than in males, we hypothesised that misidentification of the CTM would be more common in female than male subjects.

## Methods

The study was conducted following approval by the ethics and research committees of the Rotunda and Mater Misericordiae University Hospitals, Dublin. Written informed consent was obtained from patients and volunteer subjects. The Rotunda Hospital is a University-affiliated tertiary level centre for Obstetrics and Gynaecology. The Mater Misericordiae University Hospital is a tertiary level general Hospital. Recruitment took place from January to July 2013. Each trainee in the department of Anaesthesia undergoes teaching in invasive airway access using a variety of airway access devices on a manikin model at the commencement of their training rotation in this hospital. No additional training was provided prior to this study.

### Series 1

**Figure 1:**
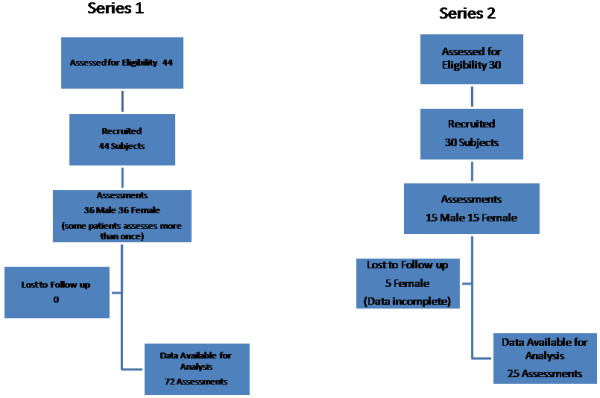
**Flow diagram.**

Using a convenience sample, 44 subjects agreed to take part in the study and consisted of 24 females and 20 Males. (Figure 1, Table 1) Both patients and members of staff acted as subjects. The 23 participants were anaesthetists who were asked to identify the CTM of the subjects. A total of 36 assessments were performed on each gender. Some of the subjects were examined more than once depending on the availability of participating anaesthetists. The median number of assessments performed per participant was 1 (IQR 1.0 – 3.0) and assessments per subject was 1 (IQR 1.0 – 2.0). 18 of the 23 anaesthetists had 3 or more years of speciality training. The methodology used has been described in detail elsewhere [9]. Briefly, the subject lay in the supine position and the CTM was identified by the assessors (MC, CMC) by palpation with ultrasonic confirmation. The limits of the CTM and the midline were marked with ink visible only on exposure to ultraviolet illumination. The participants were asked to identify the CTM by palpation with the subject’s head in the supine neutral position. The participant was asked to mark the skin overlying the CTM using the UV ink pen. The assessor then measured the distance between CTM and the estimates of the participant in both the vertical and lateral axes. A ‘correct’ estimation was defined as a mark made between the upper and lower limits of the membrane and within 5 mm of midline [9].

**Table 1:** **Series 1: subject characteristics and results**

	Female N = 24	Male N = 20	P value
Age (Yr)	41.1 (7.6)	34.8 (7.2)	<0.001
Weight (Kg)	71.0 (61.9 – 78.2)	82.1 (72.3 – 91.1)	<0.001
Height (M)	1.65 (1.65 – 1.67)	1.81 (1.74-1.82)	< 0.001
BMI (Kg.m^2^)	26.3 (24.8 – 31.9)	27.0 (23.0 – 27.8)	0.43
BMI >25 N (%)	18 (50.0)	17 (47.2)	0.98
BMI >30 N (%)	7 (19.4)	5 (13.8)	0.75
Neck Circumference (cm)	39.5 (36.5 – 40.0)	41.0 (40.5 – 42.0)	< 0.001
Participant experience (years)	10.0 (2.0 – 18.75)	5.0 (2.0 – 10.0)	0.22
Correct (N,%)	7/36	25/36	<0.001
Above (N,%)	11 (30.5)	8 (22.2)	0.59
Below (N,%)	16 (44.4)	2 (5.5)	<0.001
Distance from CTM (mm)	15.3 (10.0 – 20.4)	2.0 (0.0 – 10.8)	< 0.001
Time to identification of CTM (sec)	14.0 (12.0 – 21.0)	22.0 (15.0 -28.0)	0.11
Time to identify CTM by BMI			
BMI <25 (sec)	15.6 (3.7)	22.6 (8.9)	0.009
BMI 25-30 (sec)	16.5 (9.7)	20.8 (9.4)	0.23
BMI >30 (sec)	25.0 (16.5)	13.0 (9.2)	0.20

Categorical data are Number (%). Continuous data are mean (Standard deviation) and median (interquartile range).

### Series 2

**Figure 2:**
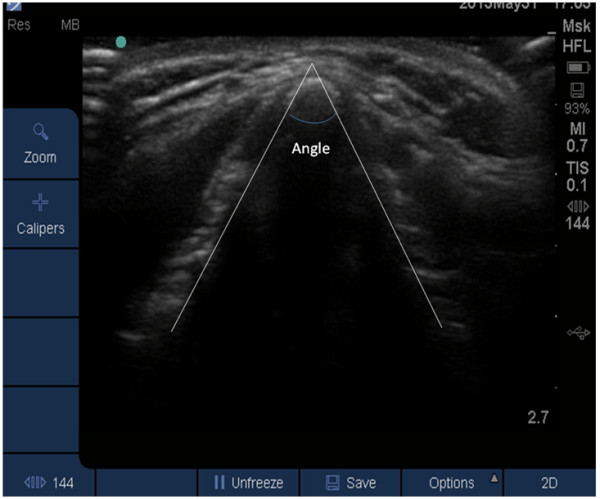
**Sample image of calculation of the angle of the junction of the thyroid cartilages.**

In a second series of 30 subjects (15 male,15 female), the thyroid cartilage was imaged using the same ultrasound equipment as in series 1 (Figure 1, Table 2). The anterior neck was imaged from caudad to cephalad using a 2.7 cm depth setting. The images were saved for offline analysis. The image which showed the maximum angulation of the thyroid was selected and printed onto paper. These were distributed to two assessors (SA, JR) blinded to subject identification and gender. The blinded assessors were asked to measure the angle of the thyroid cartilage using lines that joined the apex of the cartilage to the estimated junction of the thyroid lamina at a depth of 1 cm from the apex. (Figure 2) Participants were asked to assess the ease of palpation of the CTM using a verbal rating scale (VRS) in which 0 represented the most difficult, and 10 the easiest palpation [9].

**Table 2:** **Series 2: subject characteristics and results**

	Female N = 10	Male N = 15	P value
Age	41.0 (7.5)	36.2 (8.3)	0.12
Weight	63.6 (9.1)	84.7 (13.8)	< 0.001
Height	1.65 (0.06)	1.79 (0.06)	< 0.001
BMI	23.4 (2.9)	26.2 (3.9)	0.07
VRS palpation (cm)	8.3 (1.0)	6.2 (1.8)	0.002
Thyroid Cartilage Angle (Degree)	118.6 (9.4)	95.9 (12.9)	< 0.001
BMI >25 N (%)	5/10 (50.0)	9/15 (60)	0.7
BMI >30 N (%)	0/10 (0.0)	2/15 (13.3)	0.5
Neck Circumference (cm)	33.4 (2.6)	40.1 (1.7)	< 0.001

Categorical data are Number (%). Continuous data are mean (Standard deviation) and median (interquartile range).

### Statistical analysis

Data were analyzed using Sigma Stat (Version 2.0; Jandel Corporation, San Rafael, CA). Categorical data are presented as numbers and percentages and were analyzed by Chi-square or the Fisher exact test as appropriate. Continuous data were analyzed by the Student-*t* Test and/or ANOVA, as appropriate. Associations were determined by regression analyses. Continuous data are presented as mean (standard deviation) and median (interquartile range). In series 1, a total sample size of 72 was projected on the basis of correct identification of 60% in the male and 25% in the female subjects (α = 0.05, β = 0.8).

## Results

### Series 1

**Figure 3:**
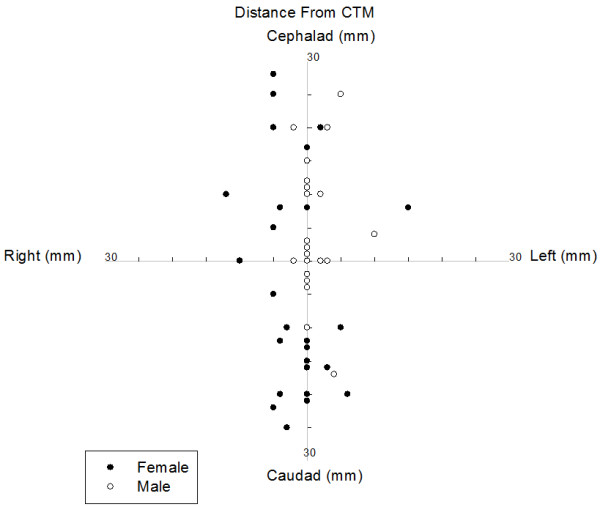
**Polar plot.** Estimation of the cricothyroid membrane position by participants. Assessment was performed with the head in the neutral position. Cepahalad and caudad markings indicate distance above or below the upper and lower limits of the cricothyroid membrane, respectively. Markings to the left and right of the vertical line indicate distance from midline.

The characteristics of the subjects are described in Table 1.The proportion of incorrectly identified CTMs was greater in female than male subjects (29/36 *vs.* 11/36, P < 0.001) (Figure 3).The deviation from the centre point of the CTM in the vertical plane was greater in females than males (11.0 [6.5 – 20.0] *vs.* 0.0 [0.0 – 10.0] mm, p <0.001). In female subjects, distance from the centre of the CTM correlated positively with neck circumference (P = 0.005) and BMI (P = 0.00005) and negatively with subject height (P = 0.01) and the experience of the assessor (P = 0.03). In male patients there was no correlation between distance from the CTM with neck circumference (P = 0.11), BMI (P = 0.3), height (P = 0.12) or experience of the assessor (P = 0.35).

### Series 2

Angulation of the posterior aspect of the thyroid cartilage was greater in females than males (118.6 ± 9.4° *vs.* 95.9 ± 12.9°, P < 0.001). This angle was lower in patients with correctly *vs.* incorrectly identified CTMs (100.0 ± 14.9° *vs.* 115.6 ± 15.9°, p = 0.02). VRS palpation was associated with increased neck circumference (P = 0.006), increased height (P = 0.002) and with decreased thyroid cartilage angle (P = 0.04). Ease of palpation was greater in correctly *vs.* incorrectly identified CTMs (VRS 7.9 ± 1.6 *vs.* 6.4 ± 1.7) and was greater in males than females (8.3 ± 1.0 *vs.* 6.2 ± 1.9, P = 0.02).

## Discussion

The data from this study show that practicing anaesthetists frequently misidentify the cricothyroid membrane and that misidentification is more frequent in female than male subjects. Furthermore, the magnitude of the error, i.e. distance from the CTM, is also greater in females than males. Within the female group the extent of error worsened with increasing BMI and neck circumference suggesting that, as might be expected, increased fat or other soft tissue obscures the anatomy and interferes with the ability of clinicians to accurately locate the CTM. This finding was not replicated in male subjects and suggests that in men increasing adiposity has a lesser effect on the ability to locate the CTM. The likely mechanism for this intergender difference is the shape of the adjacent laryngeal cartilage which is more readily identifiable because of its more acute external angulation as supported by our ultrasonic findings. The relevance of these findings is in emergency surgical airway management where misidentification of the CTM and subsequent cricothyrotomy could lead to either failure to oxygenate through malplacement or damage to surrounding vascular or airway structures [10].

Previous studies by our group and others have shown that misidentification of the CTM is common and particularly so in the female population [9, 11]. Speculative reasons for this include the similarity on palpation of the thyrohyoid space superiorly and paired tracheal rings inferiorly (more common in females), both of which may be mistaken for the CTM [10, 12]. Our finding that misidentification was more commonly below the actual CTM in females may suggest that the cricoid cartilage may be mistaken for the thyroid cartilage. Despite an obviously greater number of accurate assessments in males, the CTM was not identified correctly in 11/36 (28%) of male assessment attempts. The inability to identify the CTM in a large proportion of patients from both genders may explain at least in part the frequent failure of cricothyroid access in cadaveric laboratory experiments, prospective clinical series and meta-analysed data [5, 10, 13–15].

**Figure 4:**
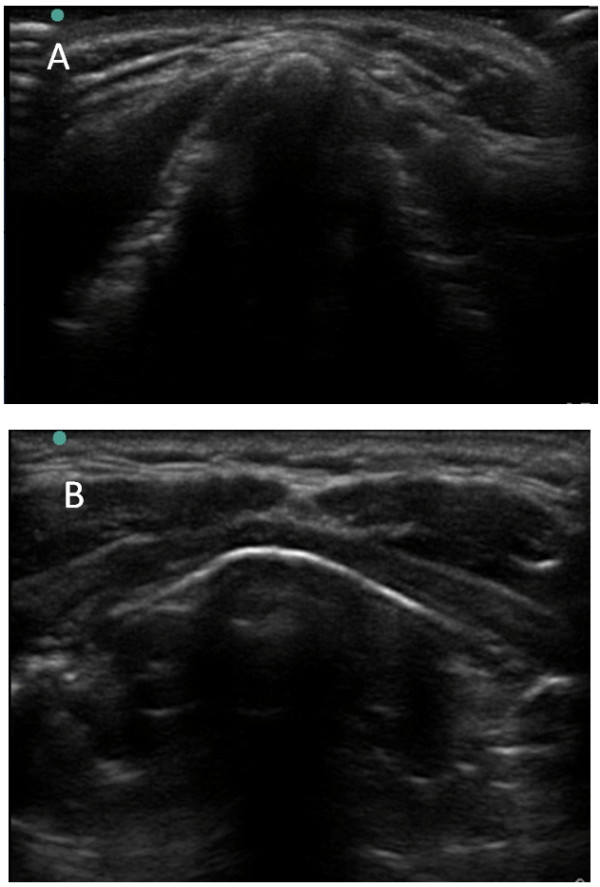
**Image of thyroid cartilage using ultrasound in the transverse plane.** The subject in panel **A** is male and is panel **B** is female.

It is intuitive that the largyngeal structures of males are more prominent than females (Figure 4). The existing literature on the subject is largely based on cadaveric dissection and goniometric measurement. Ajmani dissected the laryngeal cartilages of 28 male and 12 female adult Nigerian cadavers and found that the length, transverse diameter and anteroposterior dimensions of the larynx and the transverse and anteroposterior diameter of the cricoid cartilage were greater in male specimens [16]. Ajmani additionally demonstrated that the degree of angulation at the junction of the right and left thyroid cartilages in the midline anteriorly was 89 degrees in males and 106 degrees in females. The standard deviation of 28.3 degrees and the range of the thyroid cartilage angle in males (60 to 106 degrees) and females (88 – 132 degrees) in that series suggests that considerable anatomical variation and overlap exists in the structure within a normal population [16]. The angles observed in our study were 95.6° for males and 118.6° for females is consistent with the figures of 90° and 120° reported in cadaveric dissection of European subjects [17, 18]. Eckel reported thyroid angles in German subjects of 70.25° in males and 88.39° in females similar to that of a Swedish population [12, 19]. Angles of 85.25° and 97.85° were reported by Jain in Indian males and females respectively [20]. While confirming the intergender difference that we have demonstrated using ultrasound, these interethnic variations may have implications for accurate localization of the CTM in these populations. A further potential anatomic difference between genders that may lend itself to easier localization of the CTM in males is the consistently greater size of the adjacent cartilaginous structures which is supported by numerous cadaveric dissection studies [12, 16, 20–23].Both the vertical height and width of the CTM itself are greater in males which present a clinician with a larger target to locate [24]. Similar studies have also identified age related changes which may also have implications for locating these structures in younger subjects [25].

### Weaknesses

Our study has several weaknesses. First, we infer that if a cricothyroidotomy were performed at a misidentified site the failure rate would be equivalent to the misidentification rate. Realistically the failure rate may be higher as there is additional capacity for error due to equipment misuse or malfunction which is reflected in the high failure rate of cannula cricothyroidotomy in the NAP 4 study. Second, the study used a convenience sample and no attempt was made to randomize subjects to participants or vice versa. The number of assessments by each participating anaesthetist was not limited, and it is possible that one individual’s performance could have exerted a significant effect on the outcome of the study. No participant did more than one assessment on a single day, in an attempt to reduce any learning effect from repeated palpation. The data obtained are consistent with that obtained in a previously reported study by Aslani in females and with the cricothyroidotomy failure rate of 33% in military medicine. Third, no time limit was imposed, which does not realistically reflect the type of clinical scenario that could be anticipated. Fourth, the participants were not blinded to the gender of the subject. Fifth, all subjects were examined in the neutral anatomic position as opposed to the neck extended position which is recommended when performing a surgical airway. Last, we used the same definition of a correct marking as in Aslani’s study, which included a marking within 5 mm of the midline [9]. This is an arbitrary marking which as Aslani states may be generous. We used this measurement both for consistency of comparison and because it is likely that incisions beyond these borders might be outside the airway or may damage adjacent structures.

## Conclusions

This clearly has implications for any clinician involved in airway management and in particular in obstetric anaesthesia. The recently reported incidence of failed intubation was 1/100-1/224 in this field and these studies include a number of patients who had attempted cricothyroid access [26, 27]. Increasing BMI with pregnancy and the well documented worsening of Mallampati scores in labour may contribute to difficulty both with intubation and rescue surgical airway in this group of patients [28]. Consideration should be given to the use of ultrasound technology in CTM identification in patients who have predictably difficult anatomy [29–32]. It may be preferable to assess and mark the anatomy prior to embarking on induction of anaesthesia rather than struggling to locate the CTM as the final manoeuvre in a failed intubation algorithm when a patient is already hypoxic. Modifications of the technique of cricothyroidotomy may also be required where the anatomy is difficult. This could include an extended longitudinal incision with dissection as opposed to the ‘stab’ approach which is more appropriate when the anatomy is easily identifiable [33].
